# The House of Steinway

**Published:** 1875-05

**Authors:** 


### THE HOUSE OF STEINWAY.

"STEINWAY HALL" is, perhaps, the most delightful concert room in America. The beautiful building in which it is situated is located on East Fourteenth street, between Union Square and the Academy of Music Italian Opera House). It has a frontage of white marble, four stories high, and 50 feet wide, by a depth of 84 feet ; from this point the buildings are 100 feet wide, extending to Fifteenth street, a distance of 123 feet.

The entire first floor from Fourteenth to Fifteenth streets, a depth of 207 feet, is exclusively devoted to the exhibition and sale of the piano-fortes manufactured by the firm. At the left of the entrance on Fourteenth street is a room for Square Pianos, 17 feet high, 23 feet wide, and 84 feet deep. From this room a door leads into the house adjoining the Steinway Hall building on its westerly side (which the growing business, compelled the firm to annex), containing the office of Messrs. Steinway & Sons, from which a private telegraph extends to the Factory, two miles distant. In the rear part of this building the salesroom for the second-hand Pianos is located. Contiguous to the room for Square Pianos is the salesroom containing the Upright Pianos, from which large doors leads into the Centre Hall running through to Fifteenth street. On the easterly side of this Hall is the room devoted to Grand Pianos, which is 17 feet high, 25 feet wide, and 80 feet deep, and also two smaller rooms for the tuning and regulating of Grand Pianos. On the opposite (westerly) side of the buildings are the rooms for tuners and polishers, and the regulating room, where every Piano is carefully examined and tested in all its parts, prepared for the climate of its destination, and thoroughly regulated prior to being shipped or sent home. The main entrance to the warerooms and upper floors of the front building is through an elegant marble portico on Fourteenth street, 17 feet in width, supported by four Corinthian columns, leading to a large vestibule, from which a door on the left conducts to the warerooms, and one on the right to the ticket-office, which is located in a large vestibule with two entrances from Fourteenth street. From this latter vestibule a staircase, 14 feet wide, and from the other vestibule a staircase 7 feet wide, lead direct to a large vestibule on the next floor above, 42 feet in height, thoroughly lighted and ventilated. From this latter vestibule three large doors lead to the main floor of the Concert Hall, and two separate stairways to each of the two balconies above. The hall is 123 feet long by 75 feet wide, and 42 feet high, and has 2,000 numbered seats. The lighting by two patent sun-burner apparatuses of Defries & Son, London, is brilliant in the extreme. The hall, as well as the whole building, is heated entirely by steam, and the ventilation is most complete. The hall, with its splendid outfit and frescoing, and its boldly arched galleries, at once creates the impression that it is an opera hall, without its losing the noble simplicity of a grand concert-room ; and according to the unanimous verdict of artists, the musical public, and the newspaper press, in regard to its acoustic qualities, is admitted to surpass every other music hall in the United States. In connection with this large hall, which is supplied with an organ of forty-two registers, there is also a smaller hall, on the same floor and level, opposite the stage, 25 feet wide and 84 feet long, which, by means of colossal sliding partitions, can either be opened into the large hall or shut off from it. In this smaller hall 400 persons find comfortable accommodation.

